# Exposure to DEP Modifies the Human Umbilical Artery Vascular Resistance Contributing to Hypertension in Pregnancy

**DOI:** 10.3390/jox14020030

**Published:** 2024-04-18

**Authors:** Melissa Mariana, Amadeu M. V. M. Soares, Miguel Castelo-Branco, Elisa Cairrao

**Affiliations:** 1 Health Sciences Research Centre (CICS-UBI), University of Beira Interior, 6200-506 Covilha, Portugal; melissa.r.mariana@gmail.com (M.M.); mcbranco@fcsaude.ubi.pt (M.C.-B.); 2 Faculty of Sciences (FC-UBI), University of Beira Interior, 6201-001 Covilha, Portugal; 3 Centre for Environmental and Marine Studies (CESAM-UA), Department of Biology, University of Aveiro, 3810-193 Aveiro, Portugal; asoares@ua.pt; 4 Faculty of Health Sciences (FCS-UBI), University of Beira Interior, 6200-506 Covilha, Portugal

**Keywords:** endocrine disrupting compounds, diethyl phthalate, personal-care products, hypertensive disorders in pregnancy, human umbilical arteries, vasorelaxation, NO/sGC/cGMP/PKG signaling pathway, calcium channels

## Abstract

Hypertensive disorders in pregnancy (HDP) are the most prevalent diseases during pregnancy. In addition to the already identified risk factors, exposure to environmental contaminants has been also considered a new one. Phthalates, which are classified as priority environmental pollutants due to their ubiquitousness and endocrine disrupting properties, have been implicated in HDP in some epidemiological studies. Nevertheless, phthalates’ vascular impacts still need to be clarified. Thus, we aimed to understand the connection between phthalates exposure and the occurrence of gestational hypertension, as well as the pathway involved in the pathological vascular effects. We investigated diethyl phthalate’s (DEP) effect on the vascular reactivity of the human umbilical arteries (HUAs) from normotensive and hypertensive pregnant women. Both DEP’s nongenomic (within minutes effect) and genomic (24 h exposure to DEP) actions were evaluated, as well as the contribution of cyclic guanosine monophosphate and Ca^2+^ channel pathways. The results show that short-term exposure to DEP interferes with serotonin and histamine receptors, while after prolonged exposure, DEP seems to share the same vasorelaxant mechanism as estrogens, through the NO/sGC/cGMP/PKG signaling pathway, and to interfere with the L-type Ca^2+^ channels. Thus, the vascular effect induced by DEP is similar to that observed in HUA from hypertensive pregnancies, demonstrating that the development of HDP may be a consequence of DEP exposure.

## 1. Introduction

Complicating around 10% of all pregnancies, hypertensive disorders in pregnancy (HDP) are considered one of the leading causes of gestational death, with 18% incidence of maternal mortality [1,2]. Furthermore, they can also increase the risk of maternal stroke, pulmonary oedema, thromboembolism, placental abruption, and failure of several organs. Regarding the fetus, besides increased the risk of intrauterine death, HDP can also lead to intrauterine growth retardation and prematurity [2]. Hypertension is diagnosed when systolic blood pressure ≥140 mmHg and/or diastolic blood pressure ≥90 mmHg, and in pregnancy it can be classified as chronic hypertension (diagnosed before 20 weeks’ gestation), gestational hypertension (diagnosed after 20 weeks’ gestation), preeclampsia/eclampsia (diagnosed after 20 weeks’ gestation with proteinuria, organ dysfunction/seizures) and chronic hypertension with superimposed preeclampsia [1,2,3].

Several risk factors for the development of hypertension have been identified, from maternal overweight, advanced age (>35), sedentarism, alcohol and tobacco consumption, and familial predisposition [4], yet a new one has gained attention in the scientific community, which is the exposure to environmental contaminants [4]. This is an alarming situation, considering that these compounds are ubiquitous in the environment, with some of them being considered as priority environmental pollutants [4,5]. One example is phthalates, which are largely used in the plastic industry, but also as nonplasticizers in general products, including personal-care products [6]. Thus, considering the high use of these products, women are at greater risk of exposure to phthalates, which is of particular concern during pregnancy considering its vulnerability and sensitivity [7]. In fact, the relationship between phthalates exposure and hypertension has already been described. Epidemiological studies have suggested a positive association between increased levels of several phthalate monoesters (monobenzyl phthalate (MBzP), mono-3-carboxypropyl phthalate (MCPP), mono-isobutyl phthalate (MiBP), mono-n-butyl phthalate (MBP), and monoethyl phthalate (MEP)) with the development of HDP [7,8,9,10,11,12]. Regarding experimental studies, so far, maternal exposure to phthalates has only been related with hypertension in the offspring [13,14]. Thus, it is of extreme importance to understand the effects of phthalates on a woman’s vascular system during pregnancy.

In clinical research, the umbilical cord has been widely used to study cardiovascular diseases. Considering its availability and noninvasive collection, the human umbilical artery (HUA) presents as a suitable model to investigate vascular reactivity, and is a good source of smooth muscle cells (SMCs) [15,16]. Thus, the purpose of this study is to investigate the contribution of phthalates to the development of gestational hypertension. We analyzed DEP’s vascular effects in the HUA from women with versus without a diagnosis of hypertension. Overall, we intend to correlate phthalates with HDP and to elucidate the pathway(s) involved in the vascular effects that explain the pathological functional changes.

## 2. Materials and Methods

### 2.1. Tissue Collection

The umbilical cords were obtained from pregnant women whose parturition occurred in the “Unidade Local de Saúde Cova da Beira—ULS Cova da Beira, Covilhã” and “Unidade Local de Saúde da Guarda—ULS Guarda”. Both the tissue collection and the experimental analysis, which were carried out in the Health Sciences Research Centre of the University of Beira Interior (CICS-UBI, Covilhã, Portugal), were approved by the ethics committees of the aforementioned hospitals (ULS Cova da Beira, No. 33/2018, 18 July 2018, and ULS Guarda, No. 02324/2019, 27 February 2019). Written consent was obtained from the donor mothers before collecting the biological samples, and all the procedures were performed according to the Declaration of Helsinki.

In this study, a total of 40 umbilical cords from normotensive and hypertensive pregnant women were collected following vaginal parturition. With regard to HDP, for this protocol, only women diagnosed with gestational hypertension were included. In addition to the usual supplementation with folic acid and iron during pregnancy, hypertensive pregnant women were also treated with methyldopa (250 mg or 500 mg). Participants with any other pathology or pharmacological treatment were excluded from this study.

The samples were stored in a sterile physiological saline solution (PSS) supplemented with an antibiotic solution at 4 °C from the collection until their use (within 48 h).

### 2.2. Umbilical Arteries Isolation

The human umbilical arteries (HUAs) were dissected from the contiguous connective tissue, adipose tissue, and Wharton’s jelly, as performed in prior studies by Cairrao’s research group [17,18,19]. Then, to avoid interference from endothelium-derived substances, the tunica intima was physically removed by inserting a cotton thread into the lumen of the arteries. Afterwards, the arteries were cut into 3–5 mm rings, which were placed in Dulbecco′s Modified Eagle′s Medium/Nutrient Mixture F-12 Ham (DMEM-F12) (Sintra, Portugal) for 24 h with different concentrations of DEP for the subsequent contractility experiments.

### 2.3. Artery Tension Recordings

Two parallel stainless-steel wires were inserted in the lumen of the HUA rings and suspended within the organ bath chambers (LE01.004, Letica, Madrid, Spain) containing Krebs’ modified solution at 37 °C and continuously gassed with a carbogenic mixture (95% O_2_ + 5% CO_2_). The HUAs were equilibrated for 45 min until reaching a resting tension of 2–2.5 g, with the Krebs solution being renewed every 15 min. At the beginning of each independent experiment, the viability of the arteries was tested using serotonin (SER—1 μM), and those with a contraction of less than 1 g were excluded from the study. After this procedure, all protocols begun using three subsequent contractions with SER (1 μM), histamine (HIS—10 μM), and KCl (60 mM).

The tension was evaluated using an organ bath device, including an interface PowerLab/4SP (ML750, ADInstruments, Oxford, UK), an amplifier (ML118/D Quad Bridge, ADInstruments, Oxford, UK), four isometric transducers (TRI201, Panlab SA, Madrid, Spain), and a computerized system with PowerLab software version LabChart 5 (ADInstruments, Oxford, UK). This technique was executed as described by Cairrao, 2008 [17].

### 2.4. Vascular Reactivity Protocols

To ensure genetic variability of the data, at least 5 different HUAs were used for each protocol tested, with 2 to 3 replicates for each concentration under the different conditions analyzed. The experimental protocols were developed to understand how DEP affects the vascular response of isolated HUAs in normal and pathophysiological conditions. Thus, the obtained HUAs were divided in two groups: (1) those obtained from the normotensive pregnant women (normotensive group—NT) and (2) those from the hypertensive pregnant women (hypertensive group—HT). Both groups were analyzed for nongenomic and genomic effects of DEP.

A wide range of concentrations was selected according to DEP and its main metabolite (monoethyl phthalate—MEP) quantification in human biological samples [4,20,21,22,23], previous phthalates studies [24,25,26,27,28], and the in vitro–in vivo scaling factor [29]. This is defined as the need to have in vitro concentrations 20- to 200-fold higher than the maximum concentration observed in the human plasma in order to cause similar biological effects. Thus, considering that MEP has been detected in pregnant women at 0.77 µM in the serum [23] and between 0.167–0.249 µM in urine samples [4,22], the concentrations used in this study ranged from lower (0.001 µM) than and beyond 200-fold the maximum concentration (1000 µM).

In the first experiments, the nongenomic effects of DEP (0.001–1000 μM) were analyzed. Firstly, over the basal tension and then, three different vasoconstrictors (SER—1 μM; HIS—10 μM; KCl—60 mM) were added to the HUA rings, ending with increasing and cumulative doses of DEP (0.001–1000 μM). In these experiments, ethanol (DEP’s solvent) was used as control at the same percentage as DEP’s dilutions.

**Figure 1:**
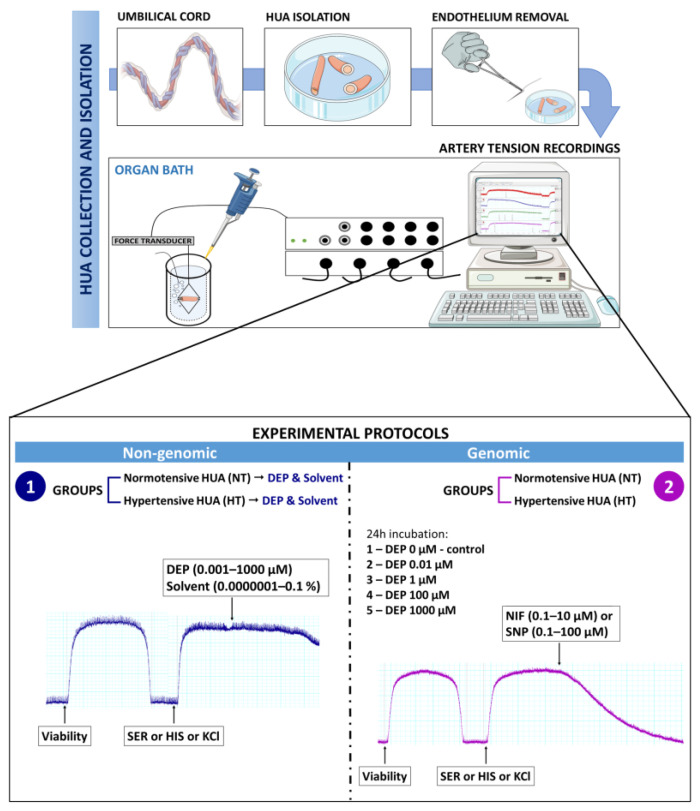
Scheme of the artery tension methods, presenting HUA isolation, tension recordings, and experimental protocols for vascular reactivity. **1** is the non-genomic experimental protocol, **2** is the genomic experimental protocol.

For the genomic experiments, the HUAs were incubated with different concentrations of DEP (0, 0.01, 1, 100, and 1000 μM) for 24 h. Afterwards, the three contractile agents chosen were added for the vasoconstriction tests to analyze and compare their potencies and efficacies upon prolonged exposure to DEP. After reaching the plateau phase, different concentrations of the vasorelaxant agents nifedipine (NIF, 0.1–10 μM) and sodium nitroprusside (SNP, 0.1–100 μM) were added to figure out the mechanism of DEP effects. Considering the photodegradable properties of SNP and NIF, the genomic studies were carried out in the absence of light. All of these protocols are schematized in Figure 1.

For both nongenomic and genomic experiments, each HUA ring was contracted with the three different vasoconstrictors, and for each experience, the contraction sequence was changed to better ensure randomness.

### 2.5. Isolation of the HUA for Smooth Muscle Cell Culture

Upon isolation of the HUAs, as described previously in Section 2 of the methods, explants from the arteries’ tunica media were placed in culture flasks of 25 cm^2^ (Orange Scientific) previously coated with collagen (5 μg/cm^2^). The explants were kept at 37 °C in a humidified atmosphere with 5% CO_2_, and the complete culture medium (HUASMC medium) was renewed every 2–3 days. Confluent cultures were attained in between 15 and 20 days, after which trypsinizations were performed to obtain subcultures that were maintained until the fourth passage, to ensure the same features as those from the living tissues regarding genetics, morphology, and electrophysiology.

All of the procedures were performed in a sterile environment with sterilized materials and solutions, as described by [17].

### 2.6. Cell Viability Determination

Cell viability was analyzed using the MTT assay, which evaluated the reduction of MTT (thiazolyl blue tetrazolium bromide—CAS nr. 298-93-1) by cellular dehydrogenases to purple formazan crystals. For this assay, subcultures of the HUASMC were performed and treated with DEP (0.001–1000 μM) dissolved in HUASMC medium followed by a 24 h incubation. A freshly mixed MTT in HUASMC medium at a concentration of 0.5 mg/mL was added to the HUASMC at 100 µL/well and incubated for 3.5 h at 37 °C, after which it was removed and replaced with DMSO (100 µL/well) to dissolve the purple formazan crystals. Then, using a microplate reader (EZ Read 400, Microplate Reader, Biochrom), the absorbance was measured at 570 nm. Ultimately, the cell viability was quantified using the following formula to calculate the percentage of viable cells relative to the control:

cell viability (%) = (Abs_sample_/Abs_control_) × 100

This procedure followed the protocol performed by Gloria et al. (2018) [18].

### 2.7. Drugs and Solutions

All chemicals and reagents were purchased from Sigma-Aldrich Química (Sintra, Portugal) except glucose, bovine serum albumin (BSA) and l-(+)-ascorbic acid (Fisher Chemical, Fisher Scientific), CaCl_2_, NaCl, EDTA and heparin (Panreac Química), KH_2_PO_4_ (ChemLab Analytical), MgCl_2_, NaHCO_3_, MgSO_4_⋅7H_2_O and KCl (Labkem), antibiotics solution (penicillin—10,000 U/mL, streptomycin—10 mg/mL, amphotericin B—25 μg/mL—PAN Biotech), and heat-inactivated fetal bovine serum (FBS—Gibco).

The solutions were stored (−20 °C) until preparation in distilled water (SER, HIS, SNP) or in absolute ethanol (DEP, NIF) according to their solubility. Appropriate dilutions were carried out in Krebs’ modified solution every day before each vascular reactivity experiment, and in the HUASMC medium for the cell viability assays. The maximum concentration of the solvent, which was used as the control, did not exceed 0.1%.

The composition of the solutions used are as follows:

- PSS (used for umbilical cord storage and isolation): CaCl_2_ (0.15 mM); KCl (5 mM); KH_2_PO_4_ (0.5 mM); NaCl (110 mM); MgCl_2_ (2 mM); NaHCO_3_ (10 mM); NaH_2_PO_4_ (0.5 mM); glucose (10 mM); EDTA (0.49 mM); HEPES (10 mM).
- Krebs’ modified solution (used for the vascular reactivity experiments): NaCl (119 mM), KCl (5 mM), CaCl_2_⋅2H_2_O (0.5 mM), MgSO_4_⋅7H_2_O (1.2 mM), KH_2_PO_4_ (1.2 mM), NaHCO_3_ (25 mM), EDTA-Na_2_ (0.03 mM), l-(+)-ascorbic acid (0.6 mM), and glucose (11 mM)—pH 7.4.
- Cell culture medium: DMEM-F12 (Dulbecco’s Modified Eagle’s Medium/Nutrient Mixture F-12 Hams) supplemented with NaHCO_3_ (1.2 g/L), L-ascorbic acid (20 mg/L), BSA (0.25%), FBS; (5%), antibiotics solution (1%), epidermal growth factor (EGF, 5 μg/mL), fibroblast growth factor (FGF, 0.5 ng/mL), heparin (2 μg/mL), insulin (5 μg/mL)—pH 7.4.

### 2.8. Statistical Data Analysis

The graphs representing the results were made using the OriginPro Graphics Software (version 9.8.0.200, 2021), with the data expressed as mean ± SEM (standard error of the mean) of the number of experiments.

The statistical assessment was performed using the SigmaStat Statistical Analysis System (version 4.0, 2008). Student’s t-test and analysis of variance (one- and two-way ANOVA) or the specific nonparametric tests were applied for the appropriate comparisons. Both the normality and homogeneity of variances were tested through Kolmogorov–Smirnov and Levene tests, respectively. A *p*-value less than 0.05 was used as criterion for determining whether the results were considered statistically significant.

## 3. Results

### 3.1. Nongenomic Effects of DEP in HUA

**Figure 2:**
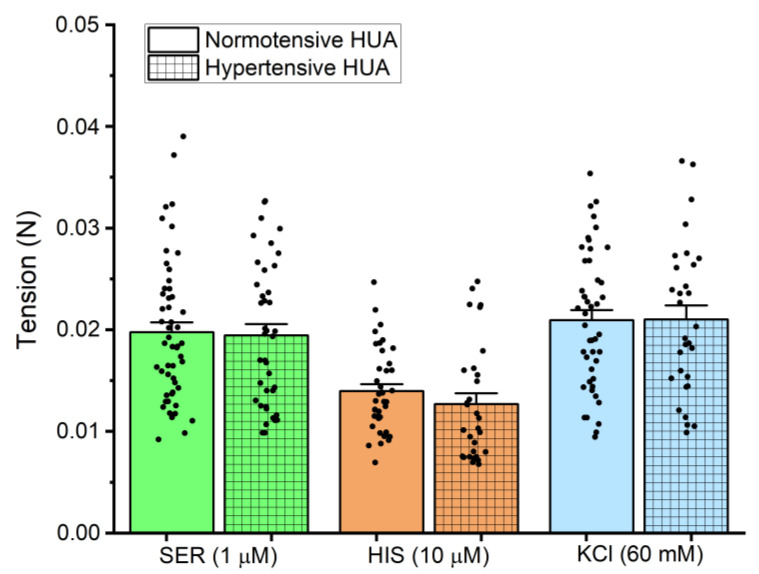
Tension (N) of HIS (10 µM), SER (1 µM), and KCl (60 mM), both in HUA rings from normotensive and hypertensive pregnant women. The columns represent the mean values, the lines the S.E.M., and the individual dots the replicates of at least five independent experiments. Statistical analysis was performed using Student’s t-test for differences between the normotensive and hypertensive groups for each contractile agent.

The vascular reactivity of the HUA was assessed using three different vasoconstrictors, two receptor agonists serotonin (SER—1 μM) and histamine (HIS—10 μM), and through isosmotic potassium chloride depolarization (KCl—60 mM). Figure 2 represents the effect of these vasoconstrictors, both in HUA rings from normotensive and hypertensive pregnant women, showing no significant differences between these two groups (*p* > 0.05).

**Figure 3:**
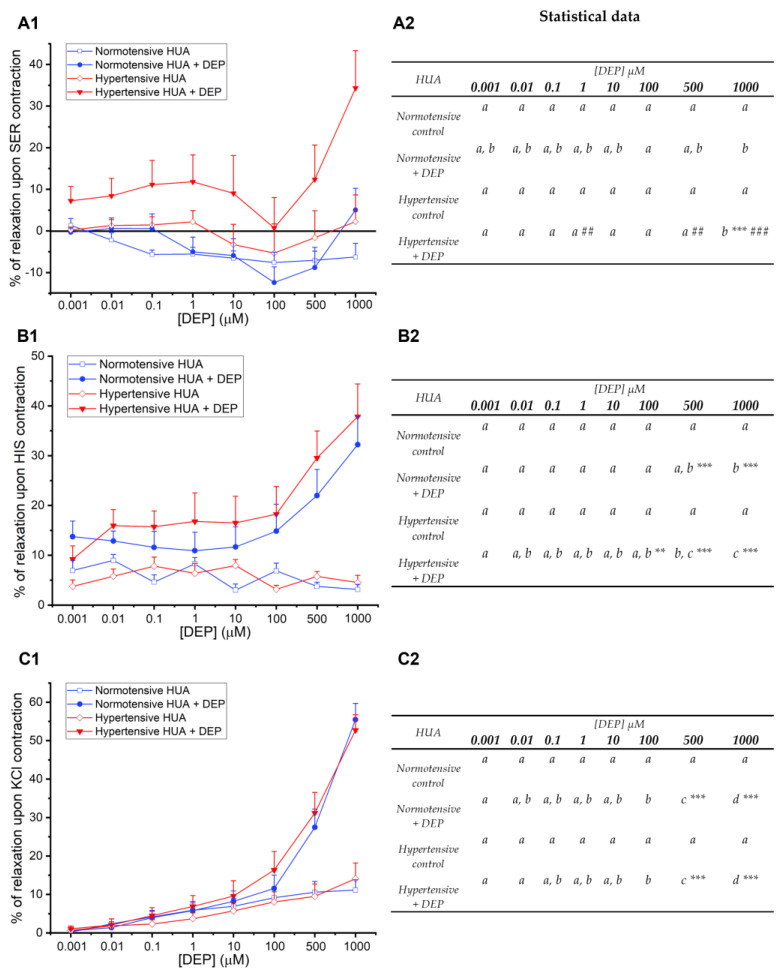
Percentage of DEP (0.001–1000 μM) vasorelaxation on HUA rings from normotensive and hypertensive pregnant women contracted with (**A1**) SER (1 µM), (**B1**) HIS (10 µM), and (**C1**) KCl (60 mM). Each point represents the mean value and the vertical lines the S.E.M. of at least five independent experiments, with two to three replicates for each concentration under different conditions. The control groups of normotensive and hypertensive HUA correspond to ethanol, used as DEP’s solvent. Data were analyzed for (**A2**) SER (1 µM), (**B2**) HIS (10 µM), and (**C2**) KCl (60 mM) contracted arteries using two-way ANOVA followed by the Holm–Sidak post hoc test. In the statistical table, different letters (a, b, c) represent significant differences between the concentrations of DEP (0.001–1000 µM) within each HUA group, * represents significant differences compared with the respective control (** *p* < 0.01; *** *p* < 0.001), and # represents differences of DEP effects between normotensive and hypertensive groups (## *p* < 0.01; ### *p* < 0.001).

Then, increasing and cumulative doses of DEP (0.001–1000 μM) were added to the contracted HUA rings, where a vasorelaxation was observed at the highest DEP concentrations (Figure 3). Specifically, after contraction with SER (Figure 3(A1,A2)), the hypertensive HUA showed a significant relaxation for DEP 1000 μM compared to all other concentrations (*p* < 0.05). There was also a significant relaxation for the hypertensive HUA group compared with the respective control and with the normotensive group, for the two highest DEP concentrations. With a different profile (Figure 3(B1,B2)), a significant relaxation was also observed for the highest DEP concentration on HIS contraction compared to all other concentrations, except for DEP 500 μM (*p* < 0.001), in normotensive and hypertensive groups. For both groups, 500 and 1000 μM DEP were significantly different from the respective control (*p* < 0.001). As seen in Figure 3(C1,C2), DEP induces a vasorelaxation in the KCl-contracted HUA in a concentration-dependent manner, being mostly significant for 500 and 1000 μM DEP compared with the remaining concentrations (*p* < 0.001), for the normotensive and hypertensive groups. When compared to the control, only the two highest DEP concentrations showed a significant relaxation (*p* < 0.001).

As expected, for the three vasoconstrictors, the solvent used to dissolve DEP had no significant effect at all percentages used, either in normotensive or hypertensive HUA. A statistical interaction between the two conditions tested (normotensive and hypertensive groups with the concentrations of DEP—*p* < 0.001) was only observed for HIS and KCl contractions. For all the contractile agents, DEP exhibited a reversible effect, considering that upon washing with Krebs’ solution, the tension returned to the basal values.

### 3.2. DEP Effects on HUASMC Viability

**Figure 4:**
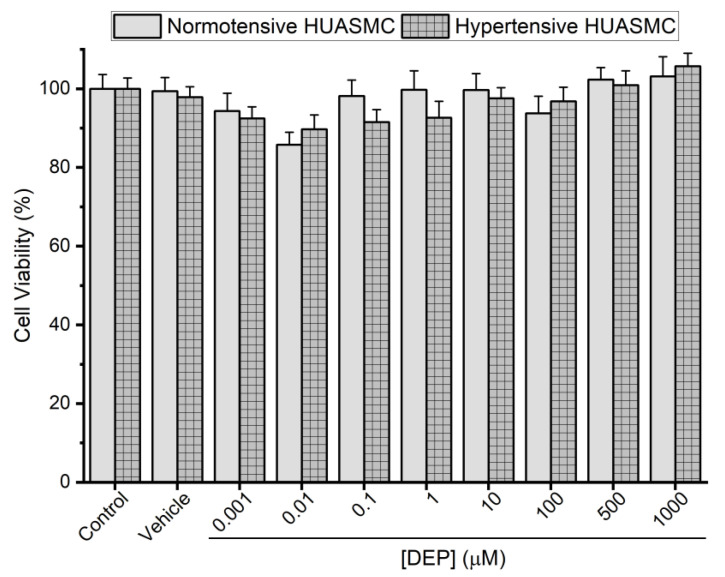
Percentage of cell viability of smooth muscle cells from normotensive and hypertensive HUA after exposure to DEP for 24 h (0.001–1000 μM). The columns represent the mean values and the lines the S.E.M. of four different experiments. Statistical assessment of concentrations of DEP vs. control and between normotensive and hypertensive groups was performed using Kruskal–Wallis followed by Dunn’s multiple comparison post hoc test.

Cultures of HUASMCs from normotensive and hypertensive pregnant women were performed to analyze the cell viability. Upon exposure for 24 h to DEP (0.001–1000 μM), to DEP solvent (ethanol at 0.1%), and to the HUASMC medium (control), the percentage of viable cells was quantified. The data obtained from the MTT assay show that the SMCs from both normotensive and hypertensive HUA presented a similar profile, as shown in Figure 4.

### 3.3. Genomic Effects of DEP in HUA

#### 3.3.1. Contractile Response

To investigate the genomic effects of DEP, the HUAs were submitted to different doses of DEP for 24 h, and then the vascular reactivity was analyzed. Four different DEP concentrations were chosen, according to the results obtained from the cell viability assays: 0.01 μM and 1000 μM, that led to the lowest and highest cell viability percentage, respectively, and two intermediate doses (1 and 100 μM).

**Figure 5:**
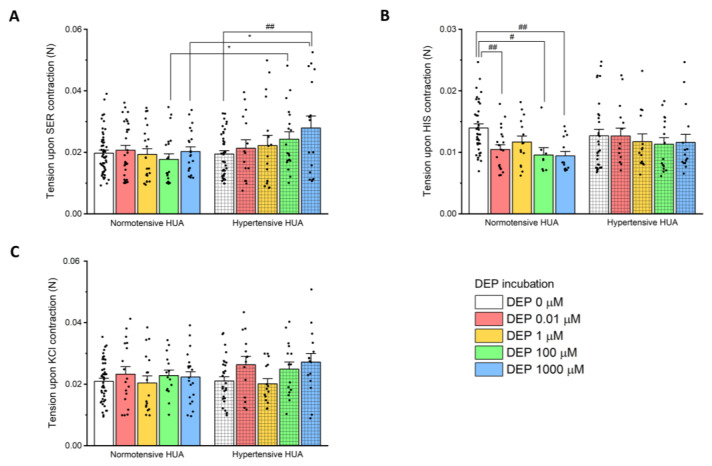
Tension (N) of normotensive and hypertensive HUA preincubated with DEP (0, 0.01, 1, 100, 1000 μM) for 24 h after contraction with (**A**) SER—1 µM, (**B**) HIS—10 µM, and (**C**) KCl—60 mM. The columns represent the mean values, the lines the S.E.M., and the individual dots the replicates of at least five independent experiments. Statistical assessment was performed using a two-way ANOVA followed by the Holm–Sidak method. * represents differences between normotensive HUA and hypertensive HUA (* *p* < 0.05), and # represents differences between DEP incubations compared to the control within normotensive HUA or hypertensive HUA (# *p* < 0.05; ## *p* < 0.01).

Figure 5 shows the HUA tension of each vasoconstrictor after 24 h DEP incubation, for both groups. As can be seen in Figure 5A, the SER tension is similar for all concentrations within the normotensive HUA group. In the hypertensive group, there was a significant difference between the arteries incubated with 1000 μM compared to the control (0 μM of DEP) (*p* < 0.01). When comparing the two groups, there was a significant difference between the normotensive and hypertensive HUA for the incubations of 100 μM and 1000 μM (*p* < 0.05).

Regarding HIS contraction, there was a significant decrease in the tension for the incubations of 0.01 (*p* < 0.01), 100 (*p* < 0.05), and 1000 μM (*p* < 0.01) when compared to the control (DEP 0 μM), but only in the normotensive HUA group.

On the other hand, similar tensions were observed in HUA contracted with KCl (Figure 5C), either in normotensive or hypertensive HUA, as well as between both groups.

#### 3.3.2. Cyclic Guanosine Monophosphate Signaling

The soluble guanylate cyclase (sGC) activation leads to increased cyclic guanosine monophosphate (cGMP) levels, leading to vasorelaxation. Thus, to understand the involvement of DEP in this pathway, different doses of SNP (a sGC activator) were added to the precontracted HUA from the normotensive and hypertensive samples, previously incubated with DEP for 24 h (0–1000 μM).

**Figure 6:**
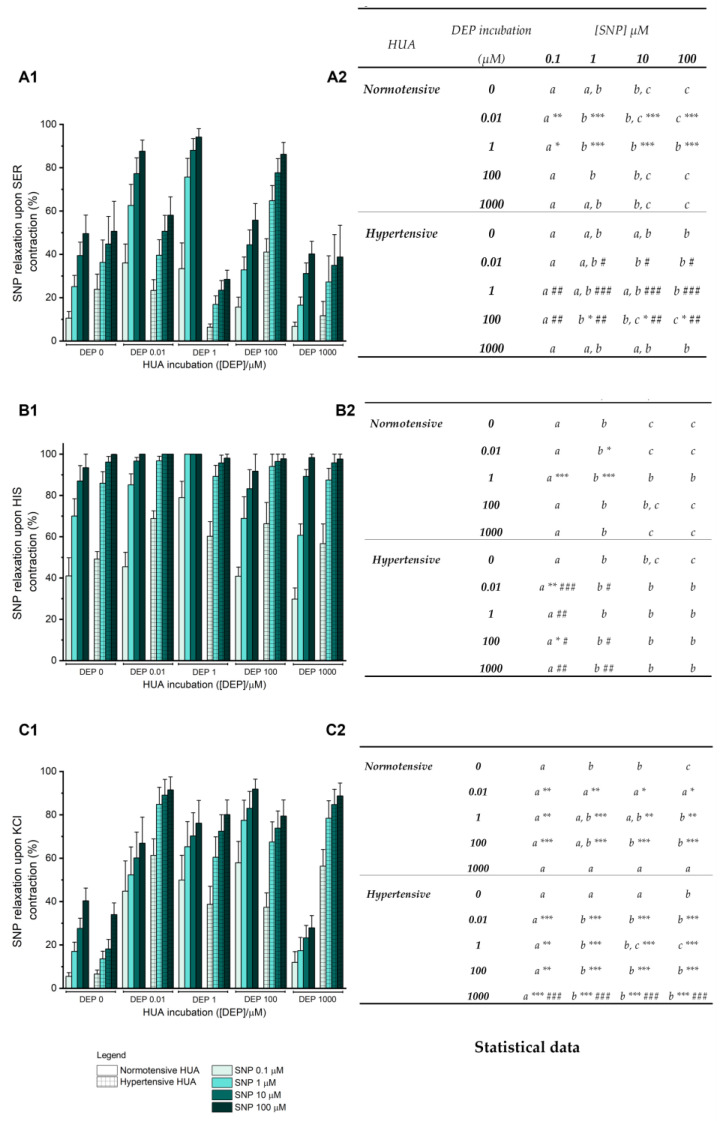
Percentage of SNP (0.1–100 μM) vasorelaxation on HUA rings from normotensive and hypertensive pregnant women preincubated with DEP (0, 0.01, 1, 100, 1000 μM) for 24 h after contracted with (**A1**) SER—1 µM, (**B1**) HIS—10 µM, and (**C1**) KCl—60 mM. The columns represent the mean values and the lines the S.E.M. of at least five independent experiments, with two to three replicates for each concentration under different conditions. The control groups of normotensive and hypertensive HUA correspond to 0 µM of DEP incubation. Data were analyzed for (**A2**) SER (1 µM), (**B2**) HIS (10 µM), and (**C2**) KCl (60 mM) contracted arteries using two-way ANOVA followed by the Holm–Sidak post hoc test. In the statistical table, different letters (a, b, c) represent significant differences between the concentrations of SNP (0.1–100 μM) within each DEP incubation, * represents significant differences compared to the control HUA (* *p* < 0.05; ** *p* < 0.01; *** *p* < 0.001), and # represents significant differences of SNP concentrations between normotensive and hypertensive HUA within each DEP incubation (# *p* < 0.05; ## *p* < 0.01; ### *p* < 0.001).

For the arteries contracted with SER (Figure 6(A1,A2)), in all DEP incubations of the two groups, increasing concentrations of SNP led to an increase in vasorelaxation. Regarding the normotensive group, the SNP relaxation in the 0.01 μM and 1 μM-incubated HUA was significantly higher in comparison to the control (DEP 0 μM) (*p* < 0.05, *p* < 0.01, and *p* < 0.001). For the hypertensive group, a significant relaxation was only observed in DEP 100 μM incubation (*p* < 0.05). When comparing both groups, there were statistical differences for DEP incubations of 0.01 μM (*p* < 0.05), 1 μM (*p* < 0.01, *p* < 0.001), and 100 μM (*p* < 0.01), with a decreased SNP vasorelaxation in the two lowest incubations for the hypertensive HUA, and an increase for the 100 μM incubation.

Figure 6(B1,B2) shows SNP effect in HIS-contracted HUA, presenting a similar profile in both normotensive and hypertensive groups; that is, increased SNP concentrations induce an increase in the vasorelaxation, with a 100% effect at the highest concentrations. In the normotensive group, there was a significant increase in the vasorelaxation of SNP 1 μM (*p* < 0.05) compared to the control in the HUA incubated with 0.01 μM of DEP. When incubated with 1 μM of DEP, this difference was observed for 0.1 and 1 μM of SNP (*p* < 0.001). In the hypertensive group, only the relaxation induced by 0.1 μM of SNP in the 0.01 and 100 μM of DEP incubation was significantly increased (*p* < 0.01 and *p* < 0.05, respectively) compared to the control. When compared with the counterpart of the normotensive group, the hypertensive group showed a significant increase in the vasorelaxant effect of SNP (0.1 μM and 1 μM) for DEP incubations of 0.01 μM (*p* < 0.001 and *p* < 0.05), 100 μM (*p* < 0.05), and 1000 μM (*p* < 0.01), whereas in the incubation of 1 μM of DEP, the effect of SNP 0.1 μM was reduced (*p* < 0.01).

In the KCl-contracted arteries, a higher vasorelaxation with increasing concentrations of SNP was shown in both groups (Figure 6(C1,C2)). The normotensive arteries incubated with 0.01 μM, 1 μM, and 100 μM of DEP presented a significantly higher relaxation when compared with HUA control for all SNP concentrations. This effect was greater the higher the DEP incubation. Similar results were found for the hypertensive HUA, with *p* < 0.001 for 1–100 μM of SNP in all DEP incubations compared to the control incubation, and for 0.1 μM of SNP, *p* < 0.01 for DEP 1 and 100 μM incubations and *p* < 0.001 for DEP 0.01 and 1000 μM incubations. When comparing both groups, there was merely a significant difference for the DEP incubation of 1000 μM, with an increased vasorelaxation for the hypertensive group in all SNP concentrations (*p* < 0.001).

#### 3.3.3. L-Type Calcium Channels Activity

Calcium channels are involved in HUA vasorelaxation, thus, to investigate the involvement of DEP in this pathway, nifedipine (NIF) was used as a Ca^2+^ channel blocker. Upon SER, HIS, and KCl contraction, NIF was added (0.1–10 μM) to the normotensive and hypertensive HUA previously incubated with DEP for 24 h (0–1000 μM).

**Figure 7:**
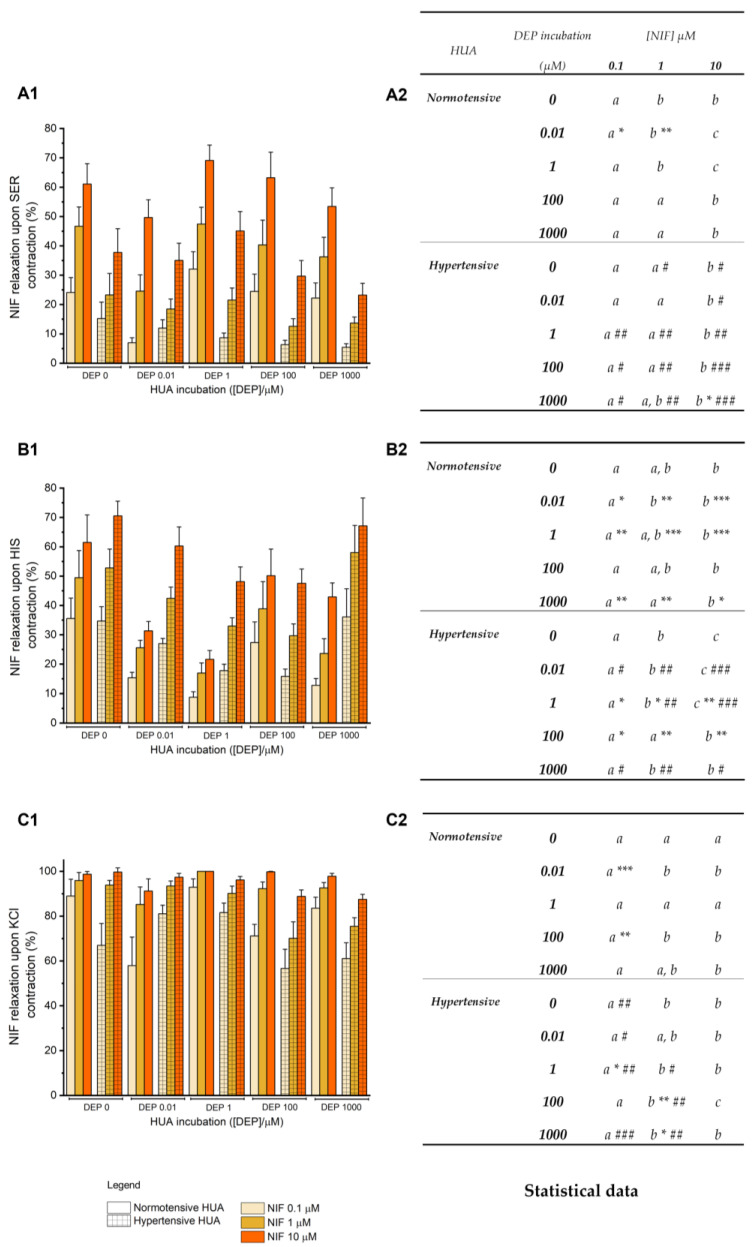
Percentage of NIF (0.1–10 μM) vasorelaxation on HUA rings from normotensive and hypertensive pregnant women preincubated with DEP (0, 0.01, 1, 100, 1000 μM) for 24 h after contracted with (**A1**) SER—1 µM, (**B1**) HIS—10 µM, and (**C1**) KCl—60 mM. The columns represent the mean values and the lines the S.E.M. of at least five independent experiments, with two to three replicates for each concentration under different conditions. The control groups of normotensive and hypertensive HUA correspond to 0 µM of DEP incubation. Data were analyzed for (**A2**) SER (1 µM), (**B2**) HIS (10 µM), and (**C2**) KCl (60 mM)-contracted arteries using two-way ANOVA followed by the Holm–Sidak post hoc test. In the statistical table, different letters (a, b, c) represent significant differences between the concentrations NIF (0.1–10 μM) within each DEP incubation, * represents significant differences compared to the control HUA (0 µM of DEP incubation) (* *p* < 0.05; ** *p* < 0.01; *** *p* < 0.001), and # represents significant differences of NIF concentrations between normotensive and hypertensive HUA within each DEP incubation (# *p* < 0.05; ## *p* < 0.01; ### *p* < 0.001).

Figure 7(A1,A2) shows the effect of HUA contracted with NIF in the normotensive and hypertensive groups, in which an increasing vasorelaxation of cumulative concentrations of NIF is apparent. When compared to the respective control, there was a significant decrease in the NIF effect (0.1 μM and 1 μM with *p* < 0.05 and *p* < 0.01, respectively) in the incubation of 0.01 μM of DEP in the normotensive group. In the hypertensive group, there was a significant decrease for the highest concentration of NIF in the incubation of DEP 1000 μM (*p* < 0.05). Overall, there was a decrease in the NIF effect on hypertensive HUA compared with the normotensive ones, significant in all DEP incubations.

Regarding the NIF effect on HIS-contracted HUA, the vasorelaxation seems to be greater with increasing NIF concentrations in both groups (Figure 7(B1,B2)). In the normotensive HUA, except for DEP incubation of 100 μM, all other incubations presented a significant vasorelaxation of NIF compared with the control. For the hypertensive group, a significant decrease in the NIF effect was only observed in DEP incubations of 1 μM and 100 μM. When analyzing both groups, a statistical difference was demonstrated in the DEP incubations of 0.01, 1, and 1000 μM, in which the hypertensive HUA showed an increase in NIF vasorelaxation when compared to the normotensive counterparts.

In the NIF effect on KCl-contracted HUA (Figure 7(C1,C2)), there was an augmented vasorelaxation with increased NIF concentrations, with the highest one presenting an effect above 90% and 87% for normotensive and hypertensive groups, respectively. In the normotensive group, between the several DEP incubations and the control, a statistical decrease in vasorelaxation was found for NIF 0.1 μM, both in DEP incubations of 0.01 μM (*p* < 0.001) and 100 μM (*p* < 0.01). Regarding the hypertensive group, statistical differences were found between DEP incubations of 1 μM, 100 μM, and 1000 μM. Specifically, there was an increase in NIF 0.1 μM vasorelaxation in the incubation of 1 μM (*p* < 0.05). An opposite effect was found for 1 μM of NIF in the arteries incubated with 100 μM and 1000 μM (*p* < 0.01 and *p* < 0.05, respectively). When comparing both groups, there were statistical differences in all DEP incubations for the lowest NIF concentrations. Except for DEP 0.01 μM, in which there was an increase in the vasorelaxation for NIF 0.1 μM (*p* < 0.05) in the hypertensive group, all other incubations exhibited a decrease in the NIF 0.1 μM effect for DEP 0 μM, 1 μM, and 1000 μM, and in the NIF 1 μM for DEP 1 μM, 100 μM, and 1000 μM.

## 4. Discussion

The development of HDP has been correlated with phthalates exposure in numerous epidemiological studies [7,8,9,10,11,12]; however, experimental studies are limited to phthalates’ effects in the offspring [13,14]. Therefore, in order to fill this gap, we investigated how phthalates contribute to the development of hypertension in pregnancy.

A significant increase in phthalate biomarker concentrations in women has raised a great concern regarding exposure to phthalates, particularly in women of reproductive age and during pregnancy, for whom the application of many cosmetics and personal care products is necessary for postpartum recovery, for reducing stretch marks and blemishes, for hydration, and even for women’s self-esteem. Furthermore, pregnancy is one of the most susceptible periods, and exposure to phthalates has already been associated with pre- and postnatal adverse outcomes [30]. Thus, we chose to analyze DEP, which is the most frequently used in this type of products [30,31]. Additionally, a former study accomplished by Cairrao’s research group analyzed DEP effects in the rat aorta, demonstrating a calcium current inhibition that may be associated with cardiovascular health disturbances [28]. The rat aorta has been one of the most used models to study vascular function and cell signaling [32]; however, animal studies may not fully mimic the human body and its reactions [33]. Therefore, we resort to human umbilical cord samples, a widely used model to study the cardiovascular system and its diseases [15,16]. It has been proven that the HUA is an adequate model to study the vascular implications of EDCs in pregnancy [18,19]. Therefore, the effects of DEP in the HUA, either from normotensive or hypertensive pregnant women, were analyzed. Considering the typical nonmonotonic dose–response curve of endocrine disruptors, in which an increase in the EDC concentration does not correspond to an increase in its effect [34,35], it has been stated that a broad range of concentrations must be used when investigating these compounds. Thus, according to the criteria previously stated, for this study, DEP concentrations ranged between 0.001 and 1000 μM.

With the aim of understanding how DEP affects gestational hypertension, we outlined two approaches of investigation: (1) the nongenomic effects, caused by a rapid HUA exposure to DEP, and (2) the genomic effects, due to prolonged exposure (24 h) of HUA to the same compound. The HUA is not innervated; therefore, it depends on the local release of vasoactive substances, including serotonin, histamine, and ions such as calcium and potassium [15,19]. In this study, we resort to three vasoactive substances to assess the vascular reactivity, and although some authors consider SER to be the most potent vasoconstrictor in HUA [15,16,36], our results demonstrated that the tensions produced by SER and KCl were similar but different from the lower one induced by HIS, for both the normotensive and hypertensive groups. These results are in accordance with previous studies regarding the HUA from normotensive pregnant women [17,36,37]; however, to our knowledge, only few studies made a comparison between the HUA of normotensive and hypertensive [38] or preeclamptic pregnant women [36]. In the first case, an analogue of thromboxane A2 was used as the contractile agent, making it difficult to compare with our results [38], and in the second study, increased K^+^ concentrations were investigated in HUA reactivity. The authors of this investigation found that preeclampsia modified the vasoreactivity of the HUA by changes of the contractile response to K^+^. These results are not in line with those obtained in our study, in which the contraction induced by KCl was similar in the normotensive and hypertensive HUA; however, it must be considered that preeclampsia has an associated pathophysiology that may lead to this difference in results.

Then, we evaluated the effect that a longer exposure to DEP has on HUA contraction. We set the incubation time to 24 h, which is considered to be sufficient for genomic alterations that lead to changes in the vascular contractile properties [19,28], and performed cell viability assays to determine the potential toxic effects of DEP. According to our results, DEP was shown not to have a toxic effect on HUASMCs, either from the normotensive or the hypertensive group, since there were no differences between DEP concentrations and the control. Based on these results, we determined four concentrations of DEP for the long-term exposure, 0.01 μM and 1000 μM, that presented the lowest and highest cell viability percentage, respectively, and two intermediate doses (1 and 100 μM). After the 24 h exposure to each of these concentrations, the contractile function of the three vasoconstrictors was analyzed. The SER response at the two highest DEP incubations (100 and 1000 μM) was greater in the hypertensive group compared to the normotensive counterpart. In addition, 1000 μM of DEP led to a significant contraction when compared to the control of the hypertensive group. The response of SER was increased, possibly due to DEP modulation of its receptors. Several SER receptors are found in the HUA, the 5-HT_1B_/5-HT_1D_ and 5-HT_2A_, which are involved in the contractile response by increasing intracellular Ca^2+^ concentration ([Ca^2+^]_i_) through inhibition of adenyl cyclase and stimulation of the PLC/IP_3_ pathway, respectively, while 5-HT_7_ leads to vasorelaxation through adenyl cyclase activation [39]. Increased levels of SER and HIS have been found to be associated with a higher sensitivity of HUA to these mediators and, consequently, with the increased vascular resistance observed in pregnancy hypertensive-related diseases, such as preeclampsia [39,40,41]. In a study where the blood metabolome of female Sprague–Dawley rats was investigated for the effects of environmental contaminants, DEP was found to increase the levels of serotonin, possibly through activation of platelets to release SER or the inhibition of monoamine oxidase A [42].

Regarding HIS contraction, incubation with 0.01 μM, 100 μM, and 1000 μM of DEP induced a significantly lower contraction in comparison to the control in the normotensive group, with no differences observed for the hypertensive one. As previously stated, HIS is involved in pregnancy-induced hypertension by increasing vascular resistance, which in the HUA may be through H_1_ receptor linkage. This Gq protein-coupled receptor leads to contraction by the PLC/IP_3_ pathway stimulation, increasing [Ca^2+^]_i_ [39]. However, the HUA expresses the H_2_ receptor, which is associated with vasorelaxation by stimulation of adenyl cyclase and consequent decrease in [Ca^2+^]_i_ [39,43]. It has been stated that although the effect of HIS is mainly due to H_1_ receptor activation, the H_2_ receptor may also be activated, leading to less powerful contractions [18]. This is in agreement with our results that, in addition to HIS being the least potent vasoconstrictor, it seems that incubation with DEP also influences this response, reducing the contractile capacity of HIS.

KCl causes vascular contraction by membrane depolarization, and Ca^2+^ influx through voltage-gated channels [15,17,36]. For the KCl-contracted HUA, our results showed no significant effect upon incubation with DEP, in both normotensive and hypertensive groups.

Results from the nongenomic effects showed that DEP presented a different relaxation profile for the three vasoconstrictors. After contraction with SER, a significant relaxation was induced by the highest concentration of DEP (1000 μM), and the overall DEP vasorelaxant effect was greater in the hypertensive group. Regarding the HIS and KCl contractions, the relaxation induced by DEP was similar between the normotensive and hypertensive groups, being only significantly different from the respective controls at the highest DEP concentrations. For all the contractile agents, the maximum vasorelaxation of DEP occurred for the highest concentration, which was greater in the KCl-contracted arteries than in those contracted with amines. These data agree with a preceding experimental study from Cairrao’s research group, in which the vasorelaxant effect of DEP on KCl-induced contraction in rat aorta was superior to that induced by noradrenaline [28]; however, they were opposite to the effects of di-(2-ethylhexyl) phthalate—DEHP, on HUA, in which the arteries contracted with HIS showed a predominant relaxation [44]. It should be noted that the mode of action of these two phthalates is possibly different, given their chemical structure and consequent molecular weight differences. DEP’s effect was similar between SER and HIS only for the hypertensive group, considering that in the normotensive arteries contracted with SER, DEP seemed to reverse this response by affecting the HUA’s ability to relax. Thus, we can hypothesize that DEP’s response depends on the vasoconstrictor and their mechanism of action (discussed above), promoting a decrease in [Ca^2+^]_i_ or an increase in K^+^. For the first time, DEP appeared to affect the HUA tonus of either normotensive and hypertensive samples, evoking an endothelium-independent vasorelaxation after SER, HIS, and KCl contractions. These data are in line with our study performed in the rat aorta, in which the short-term exposure to DEP was demonstrated to inhibit the L-type calcium current, promoting vasodilation, which can affect cardiovascular health [28].

In addition to the rapid effects, it is also important to investigate the long-term effects of EDCs due to their environmental persistence [45]. Thus, the following step was to understand how DEP promotes its vasorelaxant response upon prolonged exposure. Considering that the cGMP-PKG signaling pathway is one of the main pathways involved in HUA relaxation, the vasorelaxant effect of SNP (specific sGC activator) was analyzed after 24 h of DEP exposure. These results showed that, overall, the vasorelaxant effect of SNP is directly proportional to its concentration, i.e., the higher the concentration of SNP, the greater its action. In the SER-contracted normotensive HUA, long-term exposure to the lowest doses of DEP (0.01 and 1 μM) increased the SNP effect, while for the hypertensive HUA, the effect was only increased for 100 μM. Furthermore, when comparing the two groups, the difference in the SNP response remained. The effect of SNP in HIS-contracted HUA triggered a 100% relaxation at the highest concentrations for most DEP incubations, presenting differences in the two lowest SNP concentrations between the two groups, in which at incubations of 0.01, 100, and 1000 μM of DEP, the hypertensive arteries showed greater relaxation than the normotensive ones, while the opposite occurred at incubation of 1 μM. Regarding the KCl-contracted arteries, the cGMP-PKG signaling pathway seems to be further activated for all DEP incubations, since the SNP effect was increased compared to the control, in both groups. However, exposure to the highest concentration of DEP (1000 μM) decreased this signaling pathway activity, inducing a lower vasorelaxant response in the normotensive HUA compared to the hypertensive counterpart. Overall, except for the hypertensive HUA contracted with KCl, it seems that the arteries lose their vascular reactivity when exposed to the highest levels of DEP for 24 h. Taken together, our results suggest that the NO/sGC/cGMP/PKG signaling pathway seems to be involved in DEP’s vasorelaxation. Considering the endocrine disruption properties of DEP, it has been demonstrated that this compound can act as an estrogenic agonist, with similar estradiol effects by ERα activation [46,47,48]. Furthermore, bearing in mind that one of the mechanisms by which estrogens promote vasorelaxation is through the NO/sGC/cGMP/PKG signaling pathway, and thus promoting a decrease in blood pressure [49,50,51], we can hypothesize that DEP shares the same mechanism as estrogens in the HUA. Although there are no studies in this regard, evidence seem to suggest that there may be differences in ER expression in the HUA of women with and without hypertension, since ER expression differs from arteries and veins of the umbilical cord [52], and women with preeclampsia showed a reduction or deregulation of some ERs compared to normal pregnancies [53,54,55,56]. Thus, we can infer that ER expression levels may be decreased or deregulated in the HUA of hypertensive pregnant women compared to normotensive pregnancies.

Ca^2+^ plays a key role in the vascular response, so we used NIF, a specific blocker of the L-type Ca^2+^ channels, to analyze DEP’s involvement in the Ca^2+^ influx. Overall, NIF promoted a relaxation in all precontracted arteries, with an increased effect in those contracted with KCl. When NIF was added to the SER-precontracted HUA, DEP incubations did not seem to cause any changes in the effect of NIF when compared to the control, except for the incubation of 0.01 μM for the normotensive group and 1000 μM for the hypertensive group, in which there was a decrease in the Ca^2+^ channel’s inhibition by NIF. There was an overall decrease in the inhibitory capacity of NIF, which, according to these results, seems to be dependent on the occurrence of hypertension. In this case, the HUA from pregnant women with hypertension seems to be less responsive to the action of nifedipine. On the other hand, after contraction with HIS, the effect of NIF seems to be related to the DEP incubation, since in the HUA of the normotensive group, there was a decrease in the effect of NIF for DEP incubations of 0.01, 1, and 1000 μM, while in the hypertensive group, there was only a decrease for DEP 1 μM and 100 μM. There seems to be a nonmonotonic response of nifedipine regarding DEP incubation, since there is a nonlinear relationship between the concentration and the effect [34,35]. When comparing the two groups, there was a more pronounced loss of the NIF effect in normotensive arteries, suggesting that DEP has a greater effect in these arteries. Finally, the effect of DEP incubation on NIF relaxation was not very pronounced in normotensive arteries, whereas in hypertensive arteries, the intermediate concentration of NIF caused a decrease in vasorelaxation at higher DEP incubations (100 and 1000 μM). In general, the highest concentration of NIF caused complete vasorelaxation of the arteries with and without incubation in both groups, with differences only in the lower concentrations of NIF (0.1 and 1 μM), where most DEP incubations showed a reduced effect in hypertensive compared to normotensive HUA. These data suggest that the genomic actions of DEP appear to reduce the vasorelaxant capacity of NIF by interfering with the L-type Ca^2+^ channels. According to other investigations, one of the mechanisms stated for the development of hypertension is a deregulation of the calcium homeostasis, specifically changes in the activity of the calcium channels [19,57]. Thus, we can suggest that a prolonged exposure to DEP reduces the responsiveness of HUA to nifedipine, lowering its vasorelaxant effect and thus promoting hypertension.

Although some mechanisms for phthalates’ effects have already been proposed, these are from epidemiological studies that are subject to various constraints. Thus, for the first time, an experimental study has been able to establish a link between human exposure to phthalates and hypertensive disorders in pregnancy. Using the HUA of pregnant women without and with hypertension, we concluded that DEP has the ability to modulate the vasoconstrictor’s effect, interfering with SER and HIS receptors and [Ca^2+^]_i_, evoking an endothelium-independent vasorelaxation. As previously mentioned, increased levels of SER and HIS are related to HDP due to increased vascular resistance, and DEP seems to modulate this response. After a prolonged exposure, the results from this study showed that DEP seems to share the same vasorelaxant mechanism as estrogens in the HUA, through the NO/sGC/cGMP/PKG signaling pathway, and seems to interfere with the L-type Ca^2+^ channels, diminishing NIF vasorelaxant effects contributing to hypertension.

We are beginning to unravel the pathways involved in DEP’s vascular effects that explain the pathological functional changes of gestational hypertension. Nevertheless, more and urgent studies are needed, since pregnant women are a susceptible population that is continuously exposed to phthalates-containing products.

## 5. Conclusions

Taken together, our study reveals that the HUA from hypertensive pregnancies have decreased vascular reactivity. These data are similar to those obtained from the Ca^2+^ channels blockers after DEP exposure, in HUA from hypertensive and normotensive pregnancies. On the other hand, the vascular response to NO donors after DEP exposure seems to be potentiated, both in HUA from hypertensive and normotensive pregnancies. Thus, the results from this study demonstrate for the first time that the vascular effects induced by an EDC are similar to those observed in HUA from hypertensive pregnancies, by modulating the HUA vascular resistance, through hormonal mechanisms, and calcium homeostasis disturbances. Consequently, the development of hypertension in pregnancy may be a consequence of DEP exposure.

## Data Availability

The data presented in this study are available within the article.
